# Superior bit error rate and jitter due to improved switching field distribution in exchange spring magnetic recording media

**DOI:** 10.1038/srep27048

**Published:** 2016-06-01

**Authors:** D. Suess, M. Fuger, C. Abert, F. Bruckner, C. Vogler

**Affiliations:** 1 Christian Doppler Laboratory for Advanced Magnetic Sensing and Materials, Institute for Solid State Physics, TU - Wien, Wiedner Hauptstrasse 8-10, 1040 Vienna, Austria; 2 Institute for Solid State Physics, TU - Wien, Wiedner Hauptstrasse 8-10, 1040 Vienna, Austria

## Abstract

We report two effects that lead to a significant reduction of the switching field distribution in exchange spring media. The first effect relies on a subtle mechanism of the interplay between exchange coupling between soft and hard layers and anisotropy that allows significant reduction of the switching field distribution in exchange spring media. This effect reduces the switching field distribution by about 30% compared to single-phase media. A second effect is that due to the improved thermal stability of exchange spring media over single-phase media, the jitter due to thermal fluctuation is significantly smaller for exchange spring media than for single-phase media. The influence of this overall improved switching field distribution on the transition jitter in granular recording and the bit error rate in bit-patterned magnetic recording is discussed. The transition jitter in granular recording for a distribution of *K*_hard_ values of 3% in the hard layer, taking into account thermal fluctuations during recording, is estimated to be *a* = 0.78 nm, which is similar to the best reported calculated jitter in optimized heat-assisted recording media.

Magnetic recording at high density requires small magnetic grains or islands and narrow distribution of the material properties. In addition, intrinsic noise due to the thermal fluctuations during writing has to be minimized. This is a particular challenge in heat-assisted recording, where the write process occurs at elevated temperature^1^,^2^. In heat-assisted recording for smaller grain sizes, both (i) the fundamental jitter due to thermal fluctuations during writing as well as (ii) the *T*_c_ distributions are expected to increase.

In order to break the dilemma of designing a media that has a good writability and good thermal stability for non-heat-assisted recording, exchange spring media were proposed^3^. The original goal of exchange spring media was (i) to allow for a media that is stable, even for smaller grain sizes, and (ii) to obtain an improved switching field distribution (SFD) due to the Kondorsky dependence of the switching field^4^ as a function of field angle^3^,^5^.

Experimentally exchange spring media was first demonstrated by Wang *et al*.^6^,^7^ showing a significantly reduced coercivity by maintaining a high thermal stability. Furthermore in this work it was demonstrated the predicted lower angle dispersion of the coercivity. Soon after the first experimental realization the same group demonstrated exchange spring media on the disk substrate with soft underlayer^8^.

Since about 2007, exchange spring media have been used in current products of hard disk drives. Interestingly, the grain size in these media has not decreased. Nevertheless, exchange spring media lead to an improved signal-to-noise ratio. This can obviously not attributed to the main original goal of a small grain size. We believe the reason can be found in an improved switching field distribution in exchange spring media, which was predicted by micromagnetic simulations^9^. Experimentally, the improved switching field distribution was reported by various groups^10^,^11^.

The switching field distribution is essential in order to improve the areal density in both bit-patterned magnetic recording and magnetic recording on granular films.

A further improvement beyond two layer exchange spring media was the usage of a media design with varying anisotropy as function of the layer depth. As pointed out in ref. 12 the coercive field can be much further reduced if a graded anisotropy is used. Graded media where FePt as hard magnetic layer is used was reported experimentally by various groups^13^,^14^,^15^,^16^,^17^.

## Switching field distribution due to variation of anisotropy for fully coupled exchange spring grains

In this section, we review findings on the switching field distribution for exchange spring media and graded media in cases in which the layers with different anisotropies are fully exchange-coupled.

### Bilayer exchange spring media

In order to study in detail the improved error rate in exchange spring media, we investigate the switching field as a function of the anisotropy in the media.

In single-phase media, the switching field *μ*_0_*H*_*s*,0_ is determined according to the Stoner-Wohlfarth theory as:


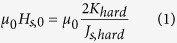


The subscript zero in the switching field denotes that in this chapter, all simulations are performed at *T* = 0 K without thermal fluctuations. Within this paper *K*_hard_ is the anisotropy of the hardest layer in the media. In the case of single-phase as it is assumed here, the media consists of only one layer. Hence *K*_hard_ denotes the anisotropy constant in the single-phase media. *J*_s,hard_ is the magnetic polarization. Using Eq. (1) we can calculate the switching field distribution, 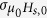, which denotes the standard deviation of the switching field at zero temperature if the anisotropy constant of the recording media (K_*hard*_) exhibits a distribution with standard deviation of *σ*_*K*,*hard*_. Since, according to Eq. (1) the switching field changes linearly with the anisotropy constant of the media, the switching field distribution 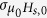 also depends linearly on the distribution of K_*hard*_:


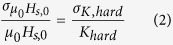


For the case of the exchange spring media, the switching field as a function of hard- or soft-layer anisotropy becomes a non-monotonic function due to the interplay between pinning field and nucleation field.

Hence, the estimate of the switching field distribution requires numerical simulations.

In the work of ref. 9, the switching field distribution of exchange spring media is investigated for uncorrelated and correlated distributions of the anisotropy constant in the soft layer and hard layer. For uncorrelated layers independent distributions of the anisotropy constant in the soft and the hard layer are assumed. The standard deviation of the anisotropy constant in the soft layer and hard layer is σ_K,soft_ = 0.1 × *K*_soft_ and σ_K,hard_ = 0.1 × *K*_hard_, respectively.

In the correlated case the ratio of the anisotropy in the soft layer and the hard layer is always the same. Hence, if for example the anisotropy in the hard layer is 5% larger than the average anisotropy in the hard layer, also the anisotropy in the soft layer is assumed to be larger by 5% compared to the average soft-layer anisotropy.

A constant mean value of the anisotropy in the hard layer is assumed. The switching field distribution is calculated for various mean values of the soft-layer anisotropy. A full coupling between the soft layer and hard layer is assumed. In ref. 9, it is concluded that for a value of the anisotropy constant in the soft layer, which is 1/5 of the anisotropy of the hard layer, the minimum switching field distribution is obtained. Due to this minimum, small variations of the anisotropy value of the soft layer or, equivalently, variations of the anisotropy constant of the hard layer do not change the switching field in the first order. Hence, the switching field distribution is significantly improved. For the case that the soft-layer anisotropy and hard-layer anisotropy are perfectly correlated, an improvement of the switching field distribution (SFD) by 35% is obtained. For the case that the layers are decoupled, the SFD is improved by 50%^9^.

### Graded media with multiple exchange coupled layers

In the case of a graded media where the anisotropy constant increases quadratically as a function of the depth of the magnetic media, the switching field no longer depends linearly as a function of the anisotropy of the hardest layer (*K*_hardest_). Using the equation as derived in ref. 12, the switching field at *T* = 0 K is given by:


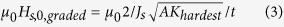


Here, *A* is the exchange constant, and *t* the layer thickness. It is assumed that within the entire grain the magnetic polarization is *J*_s_. Within this paragraph we assume that the anisotropy constants of all layers in the graded media are correlated. That means that if in the hardest layer the anisotropy is increased by a certain factor due to material fluctuation for example, in all the other layers the anisotropy is increased by the same factor. Since the switching field only varies according to 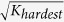 , we obtain for the switching field distribution due to a distribution of ΔK_*hardest*_:


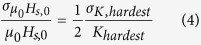


Comparing Eq. (4) with Eq. (2) one directly can observe a distinct advantage of the graded media grain. In this case (perfectly correlated layers) for the same distribution of the anisotropy constant of the hardest layer the graded media shows a switching field distribution which is smaller by a factor of two than the single-phase media.

In the case that the anisotropy of each layer of a graded media is uncorrelated and shows a distribution of 10%, the switching field distribution is given in ref. 9 to be only 3%. Hence, it is even smaller as for the correlated case, as given by Eq. (4).

To conclude, in graded media, a distribution of 10% of the *K*_hard_ values leads to a switching field distribution of 3% and 5% under the assumption of uncorrelated and correlated anisotropy constants among the layers.

## Optimizing switching field distribution by varying exchange between hard and soft layers

In contrast to ref. 9, where the exchange coupling between the soft and hard layer was kept constant and the anisotropy in the soft layer was changed, in this chapter, we investigate the switching field distribution for exchange spring media as a function of the coupling strength. Whereas full coupling will be beneficial for sufficient thick exchange spring media, decoupling the layers will be beneficial for very thin layers (thickness smaller than 10 nm), which is important, for example, for bit-patterned media.

In order to study the influence of the anisotropy on the switching field for the grain of a recording media – or, alternatively, interpret it as one island of a patterned media - we apply a field pulse for a time of 1 ns. The rise time from zero field to the maximum field is 0.1 ns. The field is applied at an angle of 10° with respect to the easy axis. The minimum field pulse strength that is required in order to switch the element is denoted as *μ*_0_*H*_s,0_.

For all investigated media, the thickness of the soft layer equals the thickness of the hard layer. In all media, the total layer thickness is 10.4 nm. The diameter of the magnetic island, which is assumed for simplicity to be faceted is *d* = 12 nm. The material parameters chosen in the paper are close to common properties for magnetic recording media such as CoCrPt media, except that a higher anisotropy is assumed in the hard layer close to values of FePt. However, it is worth noting that FePt would have a higher magnetization as the values used in this paper. The magnetic polarization as stated in refs 18,19 is about *J*_s_ = 0.5 T–0.8 T . It might be worth noting that in exchange spring media the magnetic polarization is usually designed to be slightly larger than in single-phase media. The reason is that in exchange spring structures a higher magnetic polarization can be tolerated, since the self-demagnetizing field leads to a smaller decay of the thermal stability than in single-phase media^20^. For the exchange constant within a grain a value of *A* = 10 pJ/m is assumed. The damping constant is α = 0.02, which is in the range of experimental obtained values between 0.015 and 0.024 for magnetic recording media^21^.

It is worth noting that the aim of the paper is not do evaluate the recording performance of different existing materials and composition, but rather give guidelines how a variation of certain properties such as anisotropy constants and exchange constants will affect the recording performance in particular the switching field distribution.

So we have chosen for example for the exchange spring structure, the soft-layer anisotropy constant to be exactly 1/5 of the hard-layer anisotropy constant, which is theoretically the optimum value^9^. The exchange coupling *A*_int_ between the soft layer and hard layer is varied. The exchange coupling is defined as *A*_int_ = *J S*/*a*, where *a* and *S* denote the average lattice constant and total spin quantum number in the soft and hard grain, respectively. The same value of *a* and *S* are assumed in the soft and hard layer. *J* is the exchange integral across the interface. Details on the definition of *A*_int_ can be found in ref. 22.

**Figure 1:**
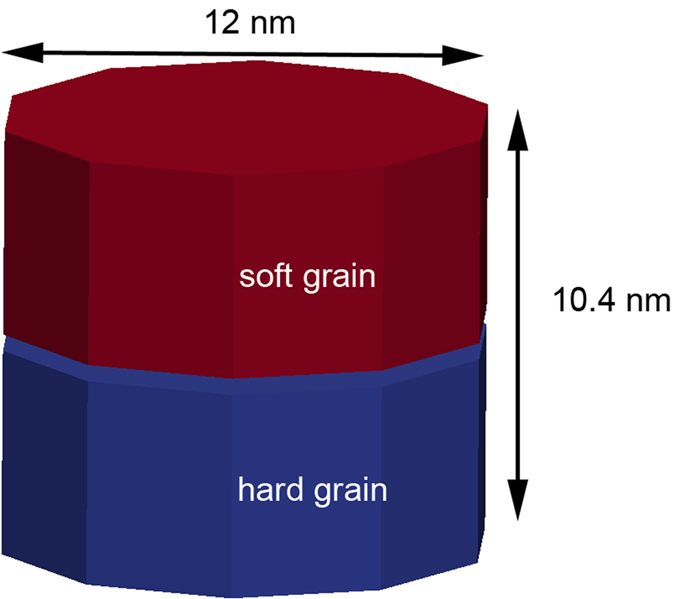
Geometry of the simulated bit-patterned island. The diameter is 12 nm, and the height is 10.4 nm. For the case of single-phase media, the blue and red grains are fully coupled. For the exchange spring structure, the coupling strength is varied.

For the exchange spring structure an atomically abrupt interface is chosen to represent a perfect two phase media. If an interface roughness is assumed in exchange spring media, the structure functionally behaves as a graded media grain^23^. The simulated geometry is shown in Fig. 1.

For the single-phase media the same geometrical model as for the exchange spring structure is used and the anisotropy is the same in the top fraction and bottom fraction of the grain. In the single-phase media there is no interface between the top layer and the bottom layer.

**Figure 2:**
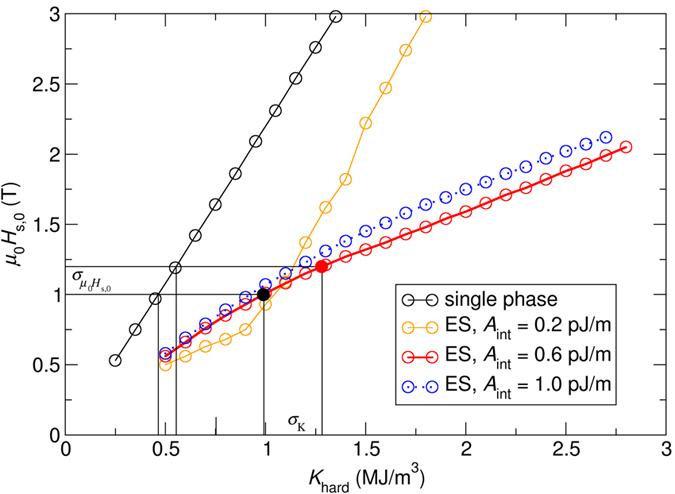
Field pulse strength, μ0Hs, 0(T) which is required to switch different media designs. The anisotropy of the media is changed.

The switching field as a function of the media layer anisotropy is plotted in Fig. 2.

The procedure as follows is used in order to extract the the correleation between switching field distribution and anisotropy distribution:

- We extract from Fig. 2 the minimal anisotropy of the hardest layer (*K*_min_), which leads to a switching field of *μ*_0_*H*_s,0,min_ = 1 T.
- We extract from Fig. 2 the maximum anisotropy of the hardest layer (*K*_max_), which leads to a switching field of *μ*_0_*H*_s,0,max_  = 1 T × 1.20.

We define the switching field variation as:


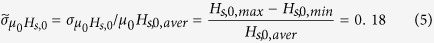



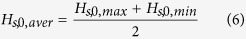


Using the procedure mentioned above, we obtain for a given switching field variation the according anisotropy variation:


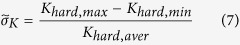


The values are summarized in Table 1. From Table 1, it can be seen that for *A*_int_ = 0.6 pJ/m and *A*_int_ = 1.0 pJ/m a larger variation of the anisotropy constant can be tolerated in exchange spring media than in the single-phase media, which leads to the same switching field variation. For single phase media and best exchange spring media 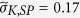 and 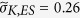 which lead to the same switching field distribution, respectively. The ratio of the anisotropy distributions which lead to the same switching field distribution is 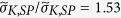.

In the following, we aim to understand the origin why a variation of the anisotropy values of the soft layer and hard layer in exchange spring media leads to a smaller change of the switching field compared to single-phase media. We investigate two different exchange spring media with different couplings between the soft and the hard layer.

### Intermediate to strong coupled case (A_int_ = 0.6 pJ/m–1.0 pJ/m)

We start with the exchange spring media design with *A*_int_ = 0.6 pJ/m, as shown by the red line in Fig. 2. The filled black dot and filled red dot represent an exchange spring media design with an anisotropy value in the hard layer of *K*_hard_ = 1 MJ/m^3^ and *K*_hard_ = 1.28 MJ/m^3^, respectively.

**Figure 3:**
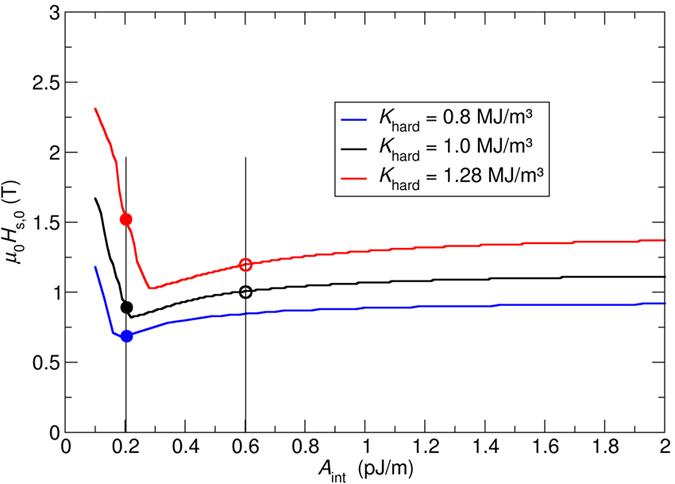
Required field pulse strength (switching field) as a function of the exchange coupling between the layers for three different values of the anisotropy of the exchange spring media.

It is important to note that the minimum of the switching pulse strength is shifted to larger values of *A*_int_ as the anisotropy is increased as it can be seen in Fig. 3. Hence, for the given exchange of *A*_int_ = 0.6 pJ/m, the media design indicated with the open red dot is closer to the minimum in the *μ*_0_*H*_s,0_ (*A*_int_) curve than the media indicated by the open black dot in Fig. 3. As a consequence, we can conclude: The increase of the anisotropy in exchange spring media will lead to the easy-to-understand increase of switching field due to higher anisotropy fields. However, since the minimum of the *μ*_0_*H*_s,0_ (*A*_int_) curve is shifted to larger values of *A*_int_ for larger anisotropy constants, this leads to an effect that reduces the switching field for larger anisotropy constants.

If the exchange constant is even further increased to *A*_int_ = *1.0 *pJ/m the switching field for this exchange constant is far away from the minimum as it can be seen in Fig. 3. Hence, in the range in which the anisotropy constant is varied (*0* < *K*_hard_ < *2 *MJ/m^3^) we expect from Fig. 3 that the switching field steadily and monotonically changes as function of the anisotropy. Indeed, this behavior can be observed as shown in Fig. 2.

### Weakly coupled case (A_int_ = 0.2 pJ/m)

**Figure 4:**
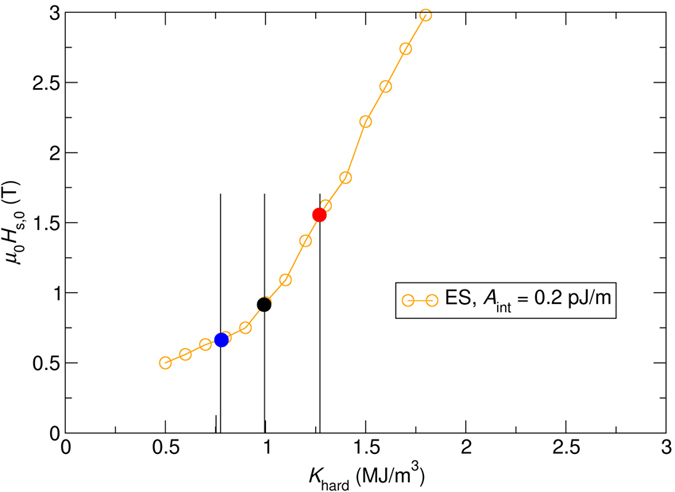
Detailed view of Fig. 2 for *A*_int_ = 0.2 pJ/m.

As a second example, we investigate a media design with *A*_int_ = 0.6 pJ/m from Fig. 2. In more detail, which is illustrated in Fig. 4. The dependence of the switching field as function of the anisotropy constant shows an even more pronounced non-linear behavior. A kink in the *μ*_0_*H*_s,0_ (*A*_int_) curve can be observed at about *K*_hard_ = 1 MJ/m^3^.

By investigating the switching field as a function of the exchange coupling between the layers for three different anisotropy constants (black dot: at the kink, blue dot: left of the kink, red dot: right of the kink), the non-linear behavior can be well understood.

The filled black dot indicating a media with *K*_hard_ = 1 MJ/m^3^ represents a design, where the exchange coupling of *A*_int_ = 0.2 pJ/m minimizes the switching field, as shown in Fig. 3. If the anisotropy of the media is decreased to *K*_hard_ = 0.8 MJ/m^3^ (solid blue dot), the same effect as mentioned in the last paragraph can be observed, which leads only to a slight reduction of the switching field.

However, for media with anisotropy values larger than *K*_hard_ > 1 MJ/m^3^, the non-linear *μ*_0_*H*_s,0_ (*A*_int_) curve leads to a significant enhancement of the switching field, as shown, for example, by the filled red dot in Figs 3 and 4. Hence, depending on the relative strength of the anisotropy field and exchange field between the layers, the non-linear *μ*_0_*H*_s,0_ (*A*_int_) curve may lead to an effect that further increases or decreases the switching field distribution.

## Effect of correlation of anisotropies between the soft and hard layer

In the previous chapter, simulations were performed, where the anisotropy in the soft layer was exactly 1/5 of the anisotropy of the hard layer. In order to also allow estimations for imperfectly correlated layers, numerical simulations are performed, where 1000 simulations with different realizations of the given distribution are averaged. The standard deviation of *K*_hard_ is assumed to be 10%.

### A. Switching field distribution for layers with correlated anisotropy

**Figure 5:**
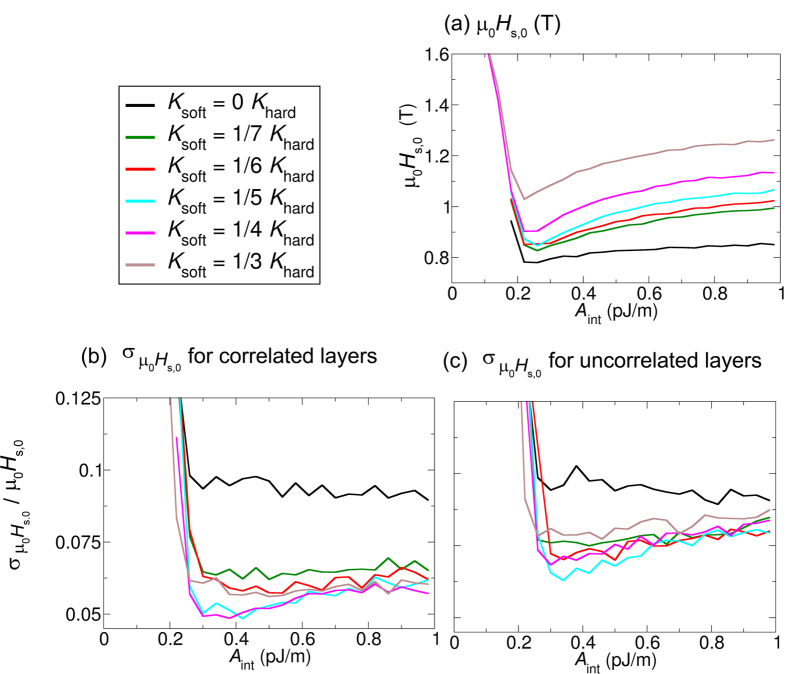
(**a**) Coercive field for soft and hard layers with correlated anisotropies. The anisotropy in the soft layer is varied. The smallest coercivity is obtained in the curve where the anisotropy of the soft layer is *K*_soft_ = 0, and the coupling is about *A*_int_ = 0.25 pJ/m. (**b**) Normalized standard deviation of the coercive field as a function of the exchange coupling between the soft and the hard layer for layers with correlated *K*_hard_. (**c**) Uncorrelated *K*_hard_. The mean value of the anisotropy of the hard layer is *K*_hard_ = 1 mJ/m^3^.

For comparison, we also perform simulations for layers with correlated anisotropy, which reproduce the results of the previous chapter for the *K*_soft_ = 1/5 *K*_hard_. In addition, we vary the anisotropy in the soft layer from *K*_soft_ = 0 to *K*_soft_ = 1/3 *K*_hard_. For all simulations, the mean value of the anisotropy of the hardest layer is *K*_hard_ = 1 MJ/m^3^. The coercive field as a function of the exchange coupling between the soft and hard layer is shown in Fig. 5(a) for a correlated layer. The cyan curve in Fig. 5(a) agrees as expected with the black curve in Fig. 3, since it is the same effect simulated with different methods.

### Switching field distribution for layers with uncorrelated anisotropy

In the following, we perform simulations for completely uncorrelated anisotropies in the soft layer and hard layer. The standard deviation of the normalized switching field (σ*μ*_0_*H*_s,0_/*μ*_0_*H*_s,0,aver_) as a function of the exchange coupling between the two layers is shown in Fig. 5(c). The smallest standard deviation of the coercive field is obtained for a ratio of the anisotropy constant between the soft layer and hard layer of 1/5 and a coupling strength between the soft layer and hard layer of about *A*_int_ = 0.35 pJ/m. Comparing Fig. 5(b,c), interestingly, the smallest standard deviation of the switching field distribution is obtained for similar values of the anisotropy constant in the correlated and uncorrelated cases.

### Comparison layers with correlated and uncorrelated anisotropies

**Figure 6:**
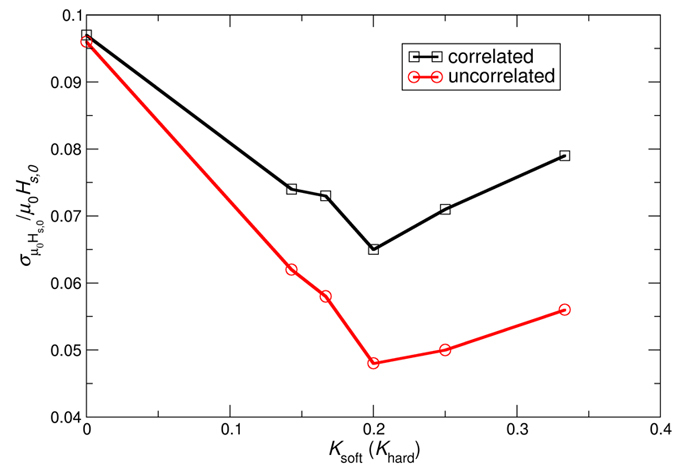
Standard deviation of the normalized switching field as a function of the anisotropy of the soft layer, which is given in units of the anisotropy of the hardest layer for *A*_int_ = 0.4 pJ/m.

The standard deviation of the coercive field as a function of the anisotropy in the soft layer for the correlated case and uncorrelated case is compared in Fig. 6. The smallest standard deviation is obtained for both cases for a value of the anisotropy constant of *K*_soft_ = 1/5 × *K*_hard_ = 0.2 MJ/m^3^. For the simulations of Fig. 6, the exchange coupling between the soft and hard layer is *A*_int_ = 0.4 pJ/m. We chose this value to be in the range of the exchange constant which gives the minimum coercive field for various anisotropy constants in the soft layer.

The smallest value of the standard deviation is obtained for the case of uncorrelated anisotropies in the layers. A correlated and uncorrelated variation of the anisotropy constant in the soft layer of 10% leads to a change of the coercive field of only about 6.5% and 4.8% for the optimized exchange spring structure, respectively.

Interestingly, if the anisotropy in the soft layer is optimized as in ref. 9 or the exchange is optimized as in this work, the switching field distribution can be reduced independently by about 35%.

The obtained results of the coercive field (Fig. 5a) and the switching field distribution (Fig. 5c,d) give interesting agreement with experimental data.

Experimentally the coercive field as well as the switching field distribution is shown as function of Pd thickness in Fig. 2 of ref. 10. Here zero Pd thickness correspond to strong coupling and 30 nm Pd thickness to weak coupling between the soft and hard layer. Hence, the *x*-axis of Fig. 2 of ref. 10 has to be inverted to be able to qualitatively compare the results with Fig. 5. A quantitative comparison is out of the scope of this paper, since it requires a detailed knowledge of the coupling strength of two layers as function of the Pd thickness.

Nevertheless, one can observe in the experimental results that there is an optimum in the coupling strength which leads to a minimum of the coercive field and a minimal switching field distribution. Even the ratio σ_SFH_/*H*_c_ which can be extracted from the data of Fig. 2 of ref. 10, which is comparable to Fig. 5(b,c) shows a minimum at an intermediate coupling strength (Pd thickness = 10 nm). Hence, the experimental data qualitatively agree well with the simulations.

## Fundamental switching field distribution due to thermal fluctuations

Besides the switching field distributions due to variations of the anisotropy values in the layers, there exists an intrinsic switching field distribution due to thermal fluctuations. This distribution occurs even for perfect grains of the same size and without any material parameter distributions and stray field interactions. This intrinsic switching field distribution can be regarded as a fundamental limiting factor for magnetic recording.

**Figure 7:**
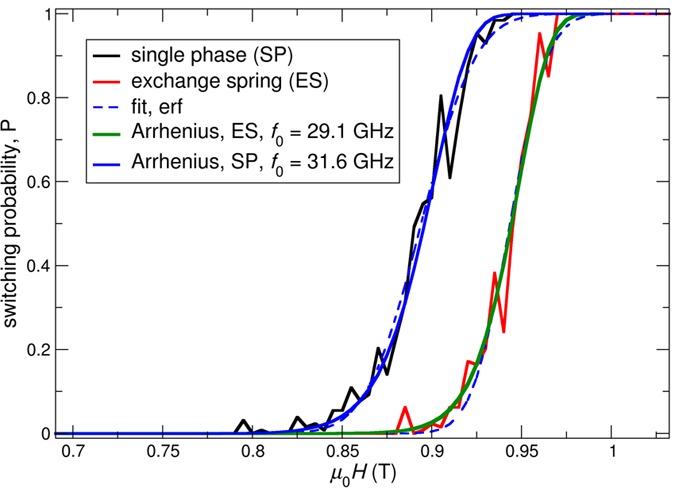
Switching field distribution due to thermal fluctuations for an exchange spring media and single-phase media at *T* = 300 K. The material parameters of the media are shown in Table 2.

In order to simulate this intrinsic distribution, the switching probability (*P*) as a function of a field pulse strength (*μ*_0_*H* (T)) is calculated for the single-phase media and the exchange spring structure of the previous section using the Landau–Lifshitz equation with a stochastic thermal field. The temperature is *T* = 300 K. The same field pulse as in the previous section is used. The field is ramped to the maximum field linearly within a time of 0.1 ns. For *t*_pulse_ = 1 ns, the field is applied with a magnitude *H*. After this time, the field is decayed to zero within 0.1 ns. In order to obtain a good statistic, each grain is simulated 128 times for the same field pulse with a different seed of the random number generator for the stochastic random field. Out of these 128 simulations for one field pulse, the switching probability is calculated. Figure 7 shows the simulated switching probability as a function of field pulse strength μ_0_*H* for the exchange spring media and the single-phase media.

In order to obtain a better understanding of the simulated switching field distributions in the following, an analytic estimate of the switching probability as a function of field pulse strength is derived. We follow the arguments of ref. 24 in order to derive the probability *P* that the particle switches, if an external field pulse of duration *t*_pulse_ and strength *H* is applied opposing the initial magnetization. The probability of switching *P* as a function of field pulse strength μ_0_ *H* is given by:


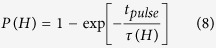


where


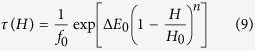


Since, in the numerical simulation, the external field is applied at a finite angle with respect to the easy axis, we assume that *n* = 1.5, which is supposed to be a good approximation for the exponent *n*^25^,^26^. The analytic switching probability according to Eq. (8) is plotted in Fig. 7. The coercive field at zero temperature *μ*_*0*_*H*_s,0_ and the energy barrier Δ*E* are obtained from independent micromagnetic simulations. The energy barrier is calculated using the nudged elastic band method^27^. The obtained parameters for *μ*_*0*_*H*_s,0_ and Δ*E* are summarized in Table 2. From a practical point of view we have assumed the same diameter of the island for single-phase media and exchange spring media, in order to make the comparison for the same areal density. Due to the higher anisotropy values in the exchange spring structure compared to the single-phase media for the same switching field, the obtained energy barrier is higher for the exchange spring structure. We have to note that we used the same finite element model for the single-phase media and exchange spring media. For the single-phase media we have assumed full coupling of the top and bottom layer, which have the same magnetic properties. Hence, the entire model represents a grain of a single-phase media and the disconnected mesh gives the same results as one mesh for the entire grain. In order to obtain good performance for the exchange spring media we reduced the coupling between the soft layer and hard layer to *A*_inter_ = 60 pJ/m.

The only free parameter is the attempt frequency *f*_0_, which is used as a fitting parameter to obtain the best agreement with the simulated results. Interestingly, the numerically obtained curves can be fitted excellently with the analytic theory according to Eq. (8), if, for both structures (exchange spring media and single-phase media), basically the same attempt frequency of *f*_0_ ~30 GHz is assumed. It is worth noting that with this single fitting parameter, not only does the switching field at *T* = 300 K (*μ*_*0*_*H*_s,300K_) agrees very well but also the slope of the *P*(*H*) curve agrees excellently.

Analyzing the analytical formula of Eq. (8), as it was done in ref. 24, an inherent advantage of the exchange spring structure appears. As it can be seen in ref. 24, the standard deviation of the switching field *σ*_c_ decreases with increasing energy barrier of the investigated structure. Hence, due to the larger energy barrier of the exchange spring structure compared to the single-phase media, the switching field distribution 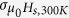
is expected to decrease.

In order to extract the thermal switching field distribution 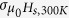 from the numerical simulated data, we assume that the derivative of the switching field follows a Gaussian function:


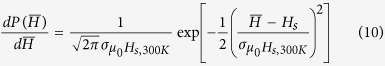


The switching probability as a function of field pulse strength is obtained by integration as follows:


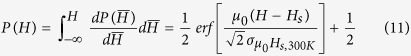


From the fit of Eq. (11) with the numerical data, we obtain 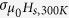 and *H*_s_, which are summarized in Table 2.

Importantly, it can be seen that indeed, the σμ_0Hs,300k_ is significantly smaller by about 33% in the case of the exchange spring media. This is also predicted by the analytical estimate according to Eq. (8), since the slope of the *P*(*H*) loop of the Gaussian fit and the analytical estimate agree excellently. The origin is the different energy barriers of the two structures.

It is worth noting that the non-Gaussian fit according to Eq. (8) is better than the fit assuming a Gaussian distribution using Eq. (11). Hence, the thermally introduced switching field distribution 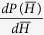 is not exactly a Gaussian function.

By comparing the obtained value of the thermal switching field distributions (Table 2) with the switching field distribution due to variations of the intrinsic anisotropy constant (Table 1), it can be seen that they are comparable in size. Hence, the thermal induced switching field distributions are an important factor, and even if σ_K_ = 0, thermal fluctuations contribute in the case of the single-phase media, with about 3% to *H*_s,300K_ distributions.

## Implication of switching field distribution for bit-patterned and granular media

### Bit error rate for bit-patterned media

**Figure 8:**
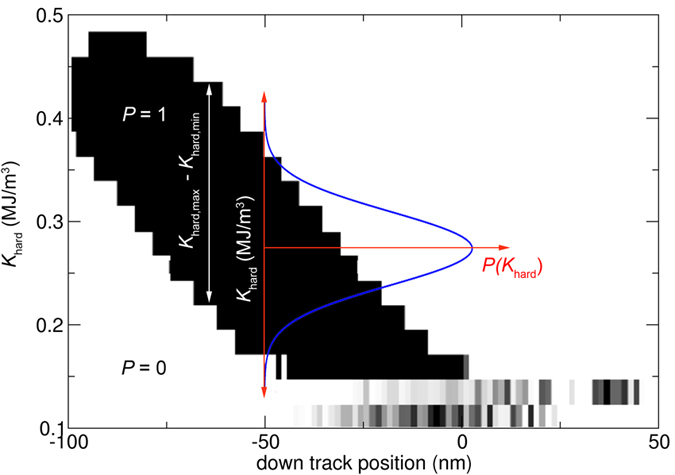
Phase plot of successful switching and non-switching for a bit-patterned media element as a function of anisotropy constant and downtrack position.

In this section, we discuss the implication of the reduced switching field distribution on the expected bit error rate (BER) of BPM. In order to derive the BER for BPM, let us assume a typical phase diagram of a bit-patterned media island as a function of downtrack position and anisotropy constant. The recording head is moved across one BPM island, and the polarity of the head is reversed from “down” to “up” and finally to “down” again. Initially, the BPM islands points in the “down” direction, which is denoted by white in the phase plot in Fig. 8. If the island is reversed to “up,” the state is denoted in Fig. 8 as black. Depending on the anisotropy constant of the island and the downtrack position of the island, the island might be able to be reversed to the “up” state. As it can be seen in Fig. 8, there is some certain interval in anisotropy constant that leads to a successful writing of the “up” state. Hence, if the distribution of the anisotropy constant is sufficiently small, there is no significant error in writing. In more detail, the BER of BPM can be calculated by assuming that the anisotropy constants are distributed according a Gaussian function, as indicated by the blue curve (Gaussian) in Fig. 8. Hence, if the tail of the Gaussian function spreads out in the region, where the islands can not be written any more, significant errors are introduced.

The BER can be calculated by integrating over the region of anisotropy constants, where successful writing is possible, and weighting it with the probability that this particular anisotropy occurs according to the Gaussian distribution:


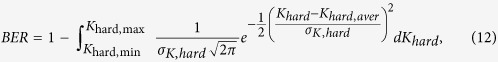


where *K*_hard,max_ and *K*_hard,min_ are the maximum anisotropy and minimum anisotropy that can be successfully written with a particular field pulse, respectively. *σ*_*K*,*hard*_ is the standard deviation of the anisotropy constant, and *K*_hard,aver_ is the average anisotropy constant. The highest BER is obtained if the media is designed such that the *K*_hard,aver_ = ½ (*K*_hard,max_ + *K*_hard,min_), which is assumed to be the case in the following. Next, we transform Eq. (12) to dimensionless variables by:


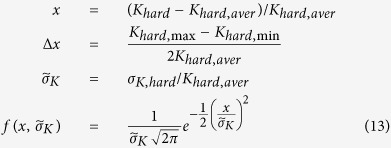


leading to:


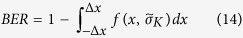


Our group showed with micromagnetic simulation for shingled recording on single-phase bit patterned media at *T* = 0 K (without strayfield interactions) and an areal density of 6.2 Tbit/inch^2^ that under the assumption of 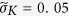 the BER is 10^−2^ ref. 28. In the studies of ref. 28 the island diameter is 8 nm, the down-track pitch is 11 nm and the cross track pitch is 9.5 nm. The thickness of the media is 12 nm and the anisotropy constant *K*_hard_ = 0.35 MJ/m^3^ and *J*_s_ = 0.7 T.

In the following we calibrate the presented semi-analytical model with the above mentioned data. We obtain the reported BER of 10^−2^ in the semi-analytical estimate for Δ*x* = 0.128:


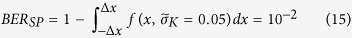


In the case of the exchange spring media at the optimized design point (for *A*_int_ = 0.6 pJ/m), the ratio of the standard deviation of the anisotropy constant of the exchange spring media and single-phase media is (using the values of Table 1) 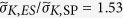. Using this data, we can interpret the exchange spring media as an single phase media with a anisotropy distribution which is reduced by a factor of 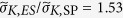. The BER for the exchange spring media is then given by:


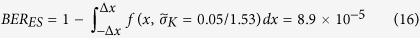


It can be seen that due to the improved switching field distribution in exchange spring media, a tremendous improvement of the BER can be expected. This tremendous increase which is derived semi - analytically here, is in agreement with micromagnetic simulations as reported in ref. 28 for exchange spring structures.

In this estimate, the stray field interaction between the islands was not taken into account. As it will be published elsewhere, the stray field interaction between islands can be regarded as an additional contribution to the switching field of about *σ*_stray_ = 0.03 T. We take the strayfield interaction into account be adding it to an effective 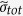 distribution as summarized in Table 3. We use this effective distribution und use Eq. (15) in order to calculate the BER for the single-phase media and exchange spring media. For the exchange spring media we use for the switching field distribution due to an intrinsic anisotropy distribution 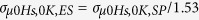, where 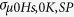, is the switching field distribution due to intrinsic anisotropy distribution of the single-phase media.

Furthermore, the distribution of the switching field due to finite temperature is taken into account using the values of Table 2, for *σ*_μ0Hs,300K_. The data is also summarized in Table 3.

If the additional distributions due to finite temperature and distributions due to strayfield are taken into account, which are assumed to be the same for exchange spring and single-phase media, an improvement of *BER* by a factor of about 2.7 and 7.6 is expected for exchange spring media for 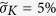 and 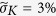, respectively (see Table 3).

### Jitter for granular media

Besides the BER for bit-patterned media, we can also estimate the jitter in granular recording. In the following, we assume a head field gradient of 0.05 T/nm. The jitter is estimated as the product of 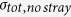 divided by the head field gradient. The results are summarized in Table 3. The total jitter value for an optimized exchange spring structure and an anisotropy distribution of 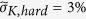 is σ_x,exchange spring_ = 0.78 nm.

For comparison an optimized heat-assisted recording single-phase media assuming 3% distribution of *T*_c_ and very narrow heat sport with a the full width at half maximum of the spatial Gaussian of the temperature profile of 20 nm leads to a cross track jitter of σ_y,heat-assists_ = 0.82 nm and a down track jitter of σ_x,heat-assists_ = 0.55 nm for a maximum write temperature of 620.0 K as reported in ref. 29. It has to be noted that in heat assisted recording besides the transition jitter which leads to AC noise also thermally written in errors are a serious problem that lead to DC error^1^,^29^. Whereas the jitter decreases in heat assisted recording if the write temperature is increased, with higher temperature DC noise increase, DC noise is expected to be a much smaller problem in non-heat assisted recording since the temperature is much smaller during writing and a higher Zeeman energy during writing helps to reverse the grain in the correct state. Basically no DC noise can be seen in Fig. 7 where 100% switching probability can be observed for sufficient large head fields.

The comparison of optimized exchange spring structure and optimized heat assisted recording single-phase media shows that the average jitter in cross track direction and downtrack direction are slightly larger than in optimized heat assisted recording but in same order of magnitude.

## Discussion

We have shown that BER in BPM can be drastically improved by an exchange spring media design. The origin of the improved BER can be found in a weak dependence of the switching field as a function of the anisotropy of both layers. This effect can be explained by the non-linear relation of the switching field as a function of the exchange coupling between the soft and the hard layer. Independently, if correlated or uncorrelated distributions of the anisotropy constant in the soft and hard layer are assumed, the minimum switching field distribution is obtained if the soft layer anisotropy is about 1/5 of the hard layer anisotropy. The switching field distribution depends significantly on the exchange coupling between the soft and the hard layer. This observation can explain the experimental obtained data of ref. 10, which also shows a minimum of the coercive field and the switching field distribution for an intermediate coupling strength between the soft and the hard layer.

As a second effect, it was shown that thermal fluctuations during recording at *T* = 300 K lead to significant additional transition jitter. This jitter increases as the thermal stability of the media decreases, leading to particular challenges for small grains at ultra-high recording density. Due to the improved thermal stability of exchange spring structure, this contribution is significantly decreased compared to single-phase media.

A simple analytical estimate is given in order to predict which improvement of BER can be expected due to the improved switching field distribution. In exchange spring media, the BER is expected to be improved by at least one order of magnitude.

The jitter contribution that is obtained due to a variation of 3% in anisotropy constant in the optimized exchange spring structure is as small as σ_x,exchange spring_ = 0.78 nm.

If this value is compare to the jitter of highly optimized heat-assisted recording media, where a jitter in downtrack direction of σ_x,heat-assists_ = 0.55 nm is reported and an higher in cross track direction, similar AC noise in the two recording schemes can be expected.

If we apply the rule of thumb that the jitter is about 20% of the minimum bit length, we obtain a bit length of 3.9 nm for the optimized exchange spring with a jitter value of 0.78 nm. Here, further studies have to be performed how smaller grain diameters influence the studies performed in this paper. Under the assumption^30^ of a bit aspect ratio of 1:3.1, the areal density can be extrapolated to be around 13 TBit/inch^2^, which is indeed very close to the original proposed areal density of 10 TBit/inch^2^ for exchange spring media^3^.

The financial support by the Austrian Federal Ministry of Science, Research, and Economy and the National Foundation for Research, Technology and Development, the SFB project F4112-N28, and the Advanced Storage Technology Consortium (ASTC) is gratefully acknowledged. The computational results presented have been achieved using the Vienna Scientific Cluster (VSC). We thank the anonymous referees for useful comments.

## Methods

Within this paper micromagnetic simulations are performed using a hybrid finite element/boundary element method^31^,^32^. In order to compress the boundary matrix that results from the boundary element method, *H*-matrices are used^33^. For simulation at zero temperature a preconditioned time integration using the backward differentiation method is applied^34^. For simulations at finite temperature a stochastic thermal field is added to the effective field of the Landau–Lifshitz equation^35^,^36^,^37^. The stochastic Landau-Lifshitz-Gilbert equation of motion is integrated using a semi-implicit method^38^.

## Additional Information

**How to cite this article**: Suess, D. *et al*. Superior bit error rate and jitter due to improved switching field distribution in exchange spring magnetic recording media. *Sci. Rep*. **6**, 27048; doi: 10.1038/srep27048 (2016).

## Figures and Tables

**Table 1:** Dependence of the anisotropy distribution which leads to a given switching field distribution of 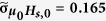 for various media designs.

	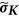	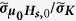
single-phase	0.17	1.02
ES, *A*_int_ = 0.2 pJ/m	0.09	1.99
ES, *A*_int_ = 0.6 pJ/m	0.26	0.68
ES, *A*_int_ = 1.0 pJ/m	0.23	0.78

**Table 2:** Thermal switching field distributions due to simulations at *T* = 300 K for grains without any intrinsic material parameter distributions.

	Single-Phase	Exchange Spring
Input:		
*d* (nm)	12	12
*t* (nm)	10.4	10.4
*K*_soft_ (MJ/m^3^)	–	0.2
*K*_hard_ (MJ/m^3^)	0.46	1.0
*J*_s_ (T)	0.7	0.7
*A*_*inter*_ (pJ/m)	1000	60
Output:		
*μ*_*0*_*H*_s,0_ (T)	0.99	1.03
*μ*_*0*_*H*_s,300K_ (T)	0.89	0.94
Δ*E* (*k*_*B*_*T*_*300*_)	113.8	155.9
*σ*_*μ0H*s,300K_ (T)	0.024	0.016
*σ*_*μ0H*s, 300K_/*μ*_*0*_*H*_s,300K_	2.7%	1.7%

**Table 3:** BER and jitter for exchange spring media and single-phase media with and without stray field interaction (*σ*_*s*tray_ = 0.03 T) for different intrinsic anisotropy distributions.

	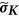	Single-Phase	Exchange Spring
*σ*_μ0Hs,0K_ (T)	5%	5 × 10^−2^	4.11 × 10^−2^
BER for *σ*_μ0Hs,0K_	5%	1.0 × 10^−2^	8.9 × 10^−5^
*σ*_x_ for *σ*_*μ*0Hs,0K_ (nm)	5%	1.0	0.65
*σ*_tot,nostray_ (T) = (*σ*_μ0Hs,0K_^2^ + *σ*_*μ*0Hs,300K_^2^)^0.5^	5%	5.5 × 10^−2^	3.6 × 10^−2^
BER for *σ*_tot,nostray_	5%	2.0 × 10^−2^	3.7 × 10^−4^
*σ*_x_ for *σ*_tot,nostray_ (nm)	5%	1.11	0.72
*σ*_tot,stray_ (T) = (*σ*_μ0Hs,0K_^2^ + *σμ*_0Hs,300K_^2^ + *σ*_stray_^2^)^0.5^	5%	6.3 × 10^−2^	5.3 × 10^−2^
BER for *σ*_tot,stray_	5%	4.2 × 10^−2^	1.6 × 10^−2^
*σ*_x_ for *σ*_tot,stray_ (nm)	5%	1.26	0.94
*σ*_tot,stray_ (T) = (*σ*_μ0Hs,0K_^2^ + *σ*_μ0Hs,300K_^2^ + *σ*_stray_^2^)^0.5^	3%	4.8 × 10^−2^	3.9 × 10^−2^
BER for *σ*_tot,stray_	3%	7.6 × 10^−3^	1.0 × 10^−3^
*σ*_x_ for *σ*_tot,stray_ (nm)	3%	0.97	0.78

The switching field distribution *σ*_μ0Hs,0K_ denotes the distribution which originates from the distribution of *K*_hard_ at *T* = 0. The influence of temperature is considered by *σ*_μ0Hs,300K_ where it is assumed that σ_K,hard_ = 0.

## References

[b1] Suess D. . Fundamental limits in heat-assisted magnetic recording and methods to overcome it with exchange spring structures. J. Appl. Phys. 117, 163913 (2015).

[b2] Wang X., Gao K.-Z., Hohlfeld J. & Seigler M. Switching field distribution and transition width in energy assisted magnetic recording. Appl. Phys. Lett. 97, 102502 (2010).

[b3] Suess D. . Exchange spring recording media for areal densities up to 10 Tbit/in(2). J. Magn. Magn. Mater. 290, 551–554 (2005).

[b4] Kondorsky E. On hysteresis in ferromagnetics. J PhysUSSR 2, 161–181 (1940).

[b5] Victora R. H. & Shen X. Composite media for perpendicular magnetic recording. Magn. IEEE Trans. On 41, 537–542 (2005).

[b6] Wang J.-P., Shen W. & Bai J. Exchange coupled composite media for perpendicular magnetic recording. IEEE Trans. Magn. 41, 3181–3186 (2005).

[b7] Wang J.-P. . Composite media (dynamic tilted media) for magnetic recording. Appl. Phys. Lett. 86, 142504 (2005).

[b8] Wang J. P., Shen W. & Hong S. Y. Fabrication and Characterization of Exchange Coupled Composite Media. IEEE Trans. Magn. 43, 682–686 (2007).

[b9] Suess D., Lee J., Fidler J. & Schrefl T. Exchange-coupled perpendicular media. J. Magn. Magn. Mater. 321, 545–554 (2009).

[b10] Hauet T. . Role of reversal incoherency in reducing switching field and switching field distribution of exchange coupled composite bit patterned media. Appl. Phys. Lett. 95, 262504 (2009).

[b11] Berger A. . Improved media performance in optimally coupled exchange spring layer media. Appl. Phys. Lett. 93, 122502 (2008).

[b12] Suess D. Multilayer exchange spring media for magnetic recording. Appl. Phys. Lett. 89, 113105 (2006).

[b13] Wang H., Zhao H., Ugurlu O. & Wang J. P. Spontaneously Formed FePt Graded Granular Media With a Large Gain Factor. IEEE Magn. Lett. 3, 4500104–4500104 (2012).

[b14] Goll D., Breitling A., Gu L., van Aken P. A. & Sigle W. Experimental realization of graded L1(0)-FePt/Fe composite media with perpendicular magnetization. J. Appl. Phys. 104, 083903 (2008).

[b15] Zhou T.-J., Lim B. C. & Liu B. Anisotropy graded FePt–TiO_2_ nanocomposite thin films with small grain size. Appl. Phys. Lett. 94, 152505 (2009).

[b16] Alexandrakis V. . Magnetic properties of graded A1/L1(0) films obtained by heat treatment of FePt/CoPt multilayers. J. Appl. Phys. 107, 013903 (2010).

[b17] Wang J. . Magnetization reversal of FePt based exchange coupled composite media. Acta Mater. 111, 47–55 (2016).

[b18] Jung H. S. . CoCrPtO-Based granular composite perpendicular recording media. IEEE Trans. Magn. 43, 2088–2090 (2007).

[b19] Jung H. S., Velu E. M. T., Malhotra S. S., Bertero G. & Kwon U. Comparison of media properties between hard/soft stacked composite and capping layer perpendicular recording media. J. Magn. Magn. Mater. 320, 3151–3156 (2008).

[b20] Suess D., Fidler J., Zimanyi G., Schrefl T. & Visscher P. Thermal stability of graded exchange spring media under the influence of external fields. Appl. Phys. Lett. 92, 173111 (2008).

[b21] Oates C. J. . High field ferromagnetic resonance measurements of the anisotropy field of longitudinal recording thin-film media. J. Appl. Phys. 91, 1417–1422 (2002).

[b22] Suess D. Micromagnetics of exchange spring media: Optimization and limits. J. Magn. Magn. Mater. 308, 183–197 (2007).

[b23] Lee J. . FePt L1(0)/A1 graded media with a rough interphase boundary. Appl. Phys. Lett. 98, 222501 (2011).

[b24] Breth L. . Thermal switching field distribution of a single domain particle for field-dependent attempt frequency. J. Appl. Phys. 112, 023903 (2012).

[b25] Victora R. H. Predicted time dependence of the switching field for magnetic materials. Phys. Rev. Lett. 63, 457–460 (1989). 10041078 10.1103/PhysRevLett.63.457

[b26] Suess D. . Reliability of Sharrocks equation for exchange spring bilayers. Phys. Rev. B 75, 174430 (2007).

[b27] Dittrich R. . A path method for finding energy barriers and minimum energy paths in complex micromagnetic systems. J. Magn. Magn. Mater. 250, L12–L19 (2002).

[b28] Suess D. & Fuger M. Bit patterned magnetic recording with and without heat assist. ASTC Review Meeting, Santa Clara, 26/9/2013. (2013).

[b29] Vogler C., Abert C., Bruckner F., Suess D. & Praetorius D. Areal density optimizations for heat-assisted-magnetic recording of high density bit-patterned media. ArXiv151203690 Cond-Mat (2015).

[b30] Ju G. . High Density Heat-Assisted Magnetic Recording Media and Advanced Characterization #x02014; Progress and Challenges. IEEE Trans. Magn. 51, 1–9 (2015).26203196

[b31] Fredkin D. R. & Koehler T. R. Hybrid method for computing demagnetizing fields. IEEE Trans. Magn. 26, 415–417 (1990).

[b32] Suss D., Schrefl T., Fidler J. & Chapman J. N. Micromagnetic simulation of the long-range interaction between NiFe nano-elements using the BE-method. J. Magn. Magn. Mater. 196, 617–619 (1999).

[b33] Forster H., Schrefl T., Dittrich R., Scholz W. & Fidler J. Fast boundary methods for magnetostatic interactions in micromagnetics. Ieee Trans. Magn. 39, 2513–2515 (2003).

[b34] Suess D. . Time resolved micromagnetics using a preconditioned time integration method. J. Magn. Magn. Mater. 248, 298–311 (2002).

[b35] García-Palacios J. L. & Lázaro F. J. Langevin-dynamics study of the dynamical properties of small magnetic particles. Phys. Rev. B 58, 14937–14958 (1998).

[b36] Lyberatos A. & Chantrell R. W. Thermal fluctuations in a pair of magnetostatically coupled particles. J. Appl. Phys. 73, 6501 (1993).

[b37] Scholz W., Schrefl T. & Fidler J. Micromagnetic simulation of thermally activated switching in fine particles. J. Magn. Magn. Mater. 233, 296–304 (2001).

[b38] Tsiantos V. . Thermal fluctuations in magnetic sensor elements. Sens. Actuators -Phys. 106, 134–136 (2003).

